# A 3D-Printable smartphone accessory for plant leaf chlorophyll measurement

**DOI:** 10.1016/j.ohx.2024.e00597

**Published:** 2024-10-24

**Authors:** Karen Ospino-Villalba, Daniel Gaviria, Daniel Pineda, Juan Pérez

**Affiliations:** a Facultad de Ciencias Agrarias. Universidad Nacional de Colombia - Sede Medellín. Carrera 65 #59a-110, Medellín, Antioquia, Colombia; b Facultad de Ciencias. Universidad Nacional de Colombia - Sede Medellín. Carrera 65 #59a-110, Medellín, Antioquia, Colombia; c Grupo SENTUS S.A.S. Medellín, Colombia; d Colcircuitos S.A.S Medellín, Colombia; e INGEOMEGA S.A.S Medellín, Colombia

**Keywords:** Smartphone sensors in plant analysis, 3D printing in agriculture, Crop sensing

## Abstract

Plant health and nutrition are universally inferred from leaf chlorophyll content. This research developed a 3D-printed accessory which attaches to the ambient light sensor in a smartphone to estimate leaf chlorophyll content in five tropical plant species. It unveils 3D printing files and assembling details to freely built the accessory anywhere. It is made from a 3D-printed body, a lighting circuit and common spare parts to measure a 663 nm LED band transmission through intact plant leaves. This chlorophyll absorbing light band allows to measure its concentration. The device was tested by comparing its readings to the universal spectrophotometric test or by leaf parallel measurements with a standard SPAD 502™ meter, and it performed as well as these universal standard methods. Due to well-studied relationships between chlorophyll concentration and nutritional status of plants, and the ubiquitous presence of other sensors in smartphones today, the independent improvement and adoption of this smartphone-connected system would ease the spread of precision farming and digital agronomy practices throughout the different scales of agriculture.

## Graphical abstract

**Figure:**
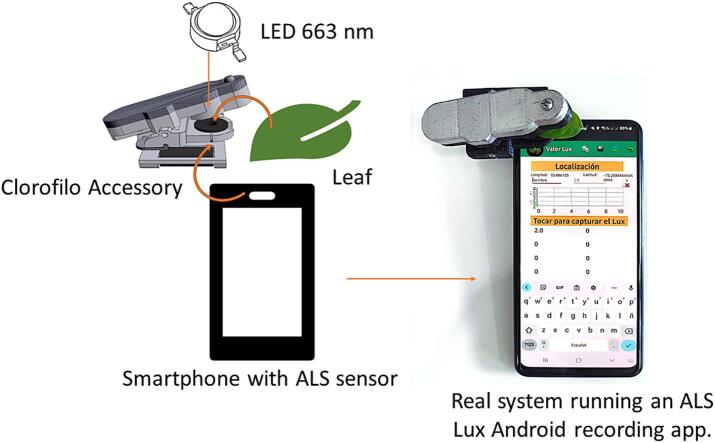

**Specifications table**.

**Table:** 

Hardware name	Clorofilo: A 3D Printed Accessory Attached to a Smartphone
Subject area	● Environmental, planetary and agricultural sciences ● Educational tools and open source alternatives to existing infrastructure ● Open-source tools ● Agricultural sciences ● Spectral properties of leaves
Hardware type	● Measuring physical properties and in-lab sensors ● Field measurements and sensors
Closest commercial analog	No commercial analog is available.
Open source license	GNU General Public License (GPL) 3.0
Cost of hardware	65,26 USD
Source file repository	https://doi.org/10.17605/OSF.IO/93WY4

## 1 Hardware in context

Leaves chlorophyll concentration is a general proxy for plant health and nutrition [1]. Chlorophyll is commonly measured by its absorption of red-light bands [2], [3] and its standard laboratory testing involves chemical leaf extraction and further optical peak-specific spectrophotometric recording for a (663 nm) and b (645 nm) chlorophylls [4], [5]. Portable chlorophyll meters based on light band transmission centered around 650 nm are also used to test chlorophyll content in intact leaves. Examples are commercial devices such as SPAD-502™ from Konica Minolta, CCM-200™ from Opti-Sciences or low-cost devices such as atLEAF + Chl meter (FT Green LLC, Wilmington, DE, USA).

Each method to measure leaf chlorophyll can have its downsides. In vitro methods are destructive, labor-intensive, time-consuming, and expensive [6]; and using portable meters can be impaired by the variable distribution of plant leaf chloroplasts [1], [7]. Although these devices can help farmers to take quick actions on crop management.

Based on knowledge about optical properties of plant leaves, consumer devices such as digital cameras alone or embedded in phones or other devices can also be adapted and used to estimate the chlorophyll content or nutritional status of plants [8], [9], [10], [11], [12], [13].

These consumer electronics might pave the way to deploy new in field analytical tools for agriculture. But these new tools should be easy to use, affordable, sustainable, and replicable anywhere. However, those promising cheap and new tools might also carry on the cost and complexity tied to needing peripheral computers or they may require external supported devices. It in turn might constrain the wide adoption of analytical systems based on those electronics, even though they have been experimentally validated [14].

Greater access to meters and autonomous use could favor the discovery of plant conditions associated with nutritional or environmental variations. Here we take advantage of the current knowledge on leaf optical properties and the widespread access to 3D printers, to build upon previous research which preliminary showed the potential use of ambient light sensors (ALS) in smartphones to measure leaf chlorophyll content [10]. Here a new and freely copy able smartphone accessory is developed and widely tested to measure light transmission through trait contrasting plant leaves and further estimate its chlorophyll content. The results indicate that this system may estimate the relative chlorophyll content in plant leaves in a way comparable to a SPAD 502™ meter and to the gold standard spectrophotometric determination of leaf chlorophyll in the laboratory.

## 2 Hardware description

The device is built using 3D printed parts from PLA (polylactic acid) filament deposition. For the sake of simplicity, it is further named Clorofilo. It works by aligning the ALS of a mobile phone to a red-light LED diode, while keeping a leaf in the middle to be measured. Leaf chlorophyll content can be further calculated as a function of leaf light transmission.

The accessory is built up from three main sections: 1) a lighting body that generates and guides light and is hinged attached to one of the walls of a U-bracket 2) the U-bracket that serves as the central platform and makes the attachment mechanism to the top front of the phone where the ALS is commonly found 3) a rotary cam cylinder on the opposite wall of the U-bracket which clamps the accessory into variable thickness phones. These sections are in turn composed of smaller glued or screwed pieces (Fig. 3) as explained in the assembly instructions.

The lighting body consists of a printed circuit board, a LED diode with a 663 nm centered band and 45 nm average bandwidth, a switch, a 100 O (Ω) protective electrical resistor, a battery stands and a 3 V coin cell battery. The hinged mechanism allows housing the electric circuit and at the same time to hold the leaf while switching on the LED diode.

The choice of this specific LED diode to measure chlorophyll through plant leaves is supported by the fact that it matches the peak of greatest red-light absorption by chlorophyll molecules in leaves around 680 nm [10], [15].

To reduce noisy light around the sensor and at the same time fix leaves without damage, a disk black rubber gasket with a 30,5mm2 inner hole is fitted facing the ALS and the LED diode.

Clorofilo analyzes chlorophyll in intact plant leaves and has functional advantages such as:

• The device can be freely manufactured anywhere.
• The size, simplicity of use and cost of the finished device can motivate its use by minimally trained operators in the field.
• It is possible to detect differences in relative chlorophyll content without calibration against standard laboratory methods by using plant leaves as living standards. This can be achieved by comparing green young leaves with senescent leaves.
• The development of custom mobile applications that use additional sensors from the smartphone such as the GPS or the camera, would enable further use in precision agriculture and digital agronomy.

**Fig. 1:**
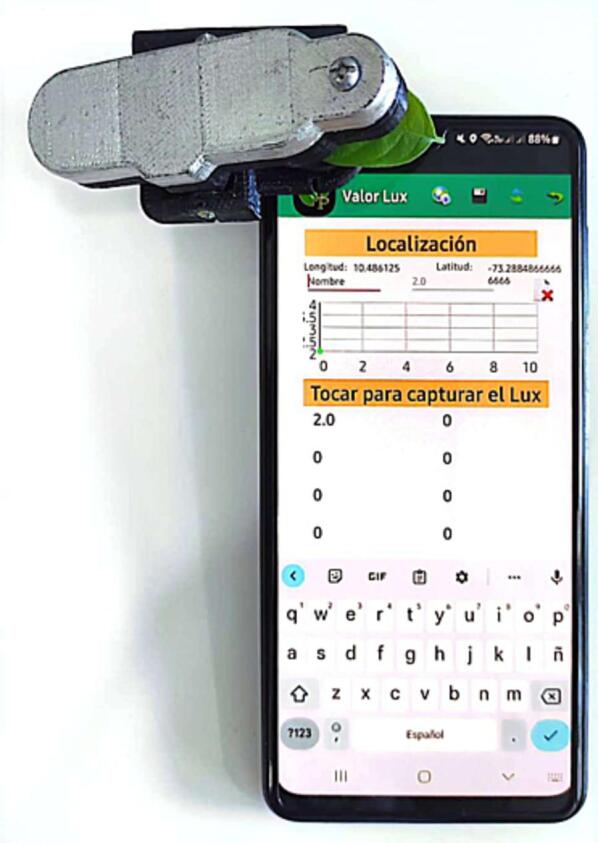
3D printed accessory installed on a smartphone running an ALS Lux recording Android application.

An image of the accessory attached to a smartphone is shown in Fig. 1. A Sony™ Xperia T2 Ultra phone was mainly used for the prototyping and testing of this accessory, although it can be adapted to mobile phone models with the ambient light sensor located at the top of the screen. These usually are located or can be hidden under the screen near the front camera.

## 3 Design files summary

**Table:** 

**Design file name**	**File type**	**Open source license**	**Location of the file**
CLOROFILO-V8tapa circuitof-1	STL file	GPL 3	https://osf.io/93wy4/ STL folder
CLOROFILO-CircuitoF3-1	STL file	GPL 3	https://osf.io/93wy4/ STL folder
CLOROFILO-V7CircuitoF3-3–1	STL file	GPL 3	https://osf.io/93wy4/ STL folder
CLOROFILO-V7Modulo3 F-VF-F-1	STL file	GPL 3	https://osf.io/93wy4/ STL folder
CLOROFILO-V7Modulo2 F-VF3-1–1	STL file	GPL 3	https://osf.io/93wy4/ STL folder
CLOROFILO-V7Modulo1 F-VF2-F1-1	STL file	GPL 3	https://osf.io/93wy4/ STL folder
CLOROFILO-V7Leva-1	STL file	GPL 3	https://osf.io/93wy4/ STL folder

• CLOROFILO-V8tapa circuitof-1: This circuit cover functions as the top piece of a housing to protect the lighting body from external agents.
• CLOROFILO-CircuitoF3-1: An intermediate spacer was designed as a stand-alone part to integrate with the circuit base, which is part of the lighting body protection housing. This division of parts prevents the formation of undesirable fillers during the 3D printing process.
• CLOROFILO-V7CircuitoF3-3–1: The circuit base is the lower piece of the lighting body and guides the LED light. It houses a common fawcett rubber gasket and also forms a screwed hinge with the U-bracket.
• CLOROFILO-V7Modulo3 F-VF-F-1: This U-bracket attaches to the lighting body and is itself fixed with two screws to the U-shaped mount. It allows the clamping mechanism to be located on the top front of the smartphone and houses an external light blocking black style C rubber grommet.
• CLOROFILO-V7Modulo2 F-VF3-1–1: An U-shaped support wedge creates space for it to snap onto the phone.
• CLOROFILO-V7Modulo1 F-VF2-F1-1: Bottom base of the U-Bracket which holds the rotary cylinder cam to adjust and immobilize the accessory on the phone.
• CLOROFILO-V7Leva-1: This rotary cylinder cam is used to immobilize the accessory to the cell phone.

## 4 Bill of materials summary

**Table:** 

**Designator**	**Component**	**Number**	**Cost per unit − USD**	**Total cost −USD**	**Source of materials**	**Material type**
Lighting circuit	Electrical Resistance (100 Ohms)	1	0,01	0,01	Local hardware store	Electronics
Lighting circuit	LED band 660 nm	1	0,75	0,75	Local hardware store	Electronics
Lighting circuit	3 V Lithium Battery	1	1,5	1,5	Local hardware store	Electronics
	3D Printed Parts	1	38	38	custom or local 3D printing service	Polymer (PLA)
	Circuit Printing	1	25	25	Local printing service	Electronics
**Total**				**65,26**		

### 4.1 Build instructions

Clorofilo was 3D modelled in Solidworks™. The pieces were built on PLA fused filament in an ordinary 3D printer. The device is composed by a lighting body, an U bracket and an eccentric cam; all built by eight smaller pieces in total (Fig. 3) as detailed below. Breaking into those smaller 8 pieces eases the printing and assemblage since no filling material needs to be cleaned.

**The lighting body** generates and guides light, while housing the assembled electric circuit. It has housing for a common faucet rubber gasket right at the LED light exit, which is intended to exclude external light and to reduce damage to the leaf to be measured. This lighting body further makes a screwed hinge with an U-bracket.

**The U-bracket** is attached to the circuit housing by a pin and tension spring. This part is made up of three pieces: piece 1 allows to install a black style C rubber grommet to allow the passage of light to the smartphonés ALS. It also isolates the sensor from external light. Piece 2 which is attached to piece 1 by two screws and dictates the space according to the smartphone thickness. And piece 3, which has two holes to house a cam cylinder.

Finally, the **eccentric cam cylinder** is attached to U-bracket and provides the necessary tightening for the accessory to the smartphone once the LED light stream and ALS sensor are aligned.

**Fig. 2:**
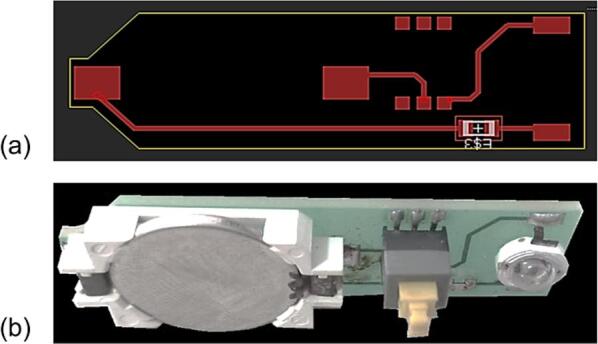
Design of the lighting circuit. (a) Digital design of the PCB circuit (b) Printed and assembled circuit with a coin battery, battery holder, a switch, a resistor, and the LED diode.

**Fig. 3:**
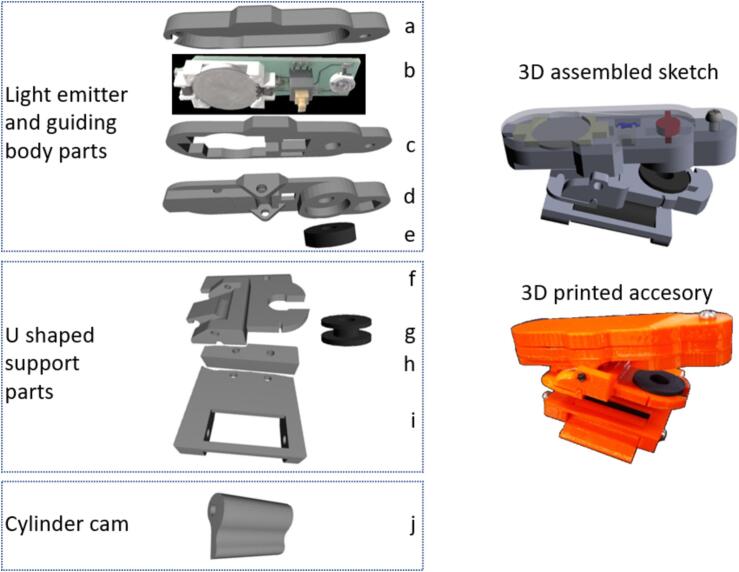
Overview and 3D printing design of the pieces making the three main sections of Clorofilo accessory. Following in parentheses are names of print or hardware repository files: a. Circuit cover (CLOROFILO-V8tapa circuitof-1). b. Electrical circuitry (Fig. 2a). c. Intermediate spacer (CLOROFILO-CircuitoF3-1). d. Circuit base (CLOROFILO-V7CircuitoF3-3–1). e. Rubber gasket (From a household water faucet). f. Top side U-bracket (CLOROFILO-V7Modulo3 F-VF-F-1). g. Black style C rubber grommet. h. U-shaped support wedge (CLOROFILO-V7Modulo2 F-VF3-1–1). i. Bottom base of the U-Bracket (CLOROFILO-V7Modulo1 F-VF2-F1-1). j. Rotary cylinder cam (CLOROFILO-V7Leva-1). The top right section shows the 3D assembled sketch, while the bottom right section displays the 3D printed accessory.

Printed parts are assembled as shown in Fig. 3. The battery, switch, 100 Ω resistor and the LED diode are soldered to the circuit as shown in Fig. 2. This circuit is housed inside the lighting body. Parts b and c are glued by any appropriate bonder. Pieces a-d are secured with a 1 cm screw and together with e form the full lighting body. This body also makes a hinge when attached with a common screw to part f in the U bracket. The U-bracket in turn is assembled with two screws and the rotating cylinder cam j is secured in part i of the U-bracket using another 3 mm diameter 5 cm long screw.

### 4.2 Operation instructions

The accessory must be used on a smartphone that houses an ALS sensor. This sensor is usually located at the front top in the screen (Fig. 1).

*Clorofilo accessory installation and mobile apps for ALS sensor reading*.

a. Clamp the assembled accessory into your smartphone.

b. Install any mobile application to read the light intensity reading by the ALS sensor (e.g., Physics Tool Box sensor Suite. Available free on:

https://play.google.com/store/apps/details?id = com.chrystianvieyra.physicstoolboxsuite.

c. Align the light path between the accessory and the smartphone ALS sensor. Check this by slightly displacing the accessory with the LED on while running the app, until a maximum lux value is shown.

*Measuring light transmission through plant leaves*.

a. Open the app and select the light intensity meter option.
b. Insert leaf samples bypassing the central vein.
c. Press the clamp until the switch is turned on.
d. Record your light transmission values through the leaf.

### 4.3 Validation of the CLOROFILO accessory readings against standard chlorophyll measurement systems

The performance of the Clorofilo accessory was tested in two separate experiments. The first one used a set of 86 fresh plant leaves detached from five common tropical species, to compare on a same spot its Clorofilo readings to the spectrophotometric determination of chemically extracted chlorophyll (see details on next section). The second experiment made parallel readings at the same spots of coffee leaves by using the Clorofilo accessory and a SPAD 502™ meter.

For experiment 1 the results are presented as scatterplot of light transmission values and spectrophotometric chlorophyll concentration readings in five plant species. Experiment 2 presents parallel Clorofilo light transmission values and SPAD 502™ indexes in coffee leaves. Adjusted regression models and coefficients of determination were obtained on the Statlet tool of the statistical program Statgraphics® centurion 19. Details of those experiments are presented next. Light transmission values are presented form this point forward as cLux values, instead of common lux values. This differentiate cLux units which comes from a red-light exciting band, from common lux values which are regularly calculated from red, green and blue exciting light bands.

***Light transmission measurement and chlorophyll spectrophotometric determination in leaves of five tropical plant species***.

A set of 86 leaves was collected in the surroundings of the National University of Colombia on its Medellín campus (Colombia) and was composed by 18 leaflets of oil palm (*Elaeis guineensis; Jacq*), 15 potato leaves (*Solanum tuberosum; L.*), 15 coffee (*Coffea arabica L*.), 14 cocoa (*Theobroma cacao*) and 24 kikuyu grass (*Pennisetum clandestinum*) leaves. Those were visually selected by their apparent differences in green color intensity which might ensure a wide range of LCC. These fresh leaves were wrapped in wet paper towels, inserted into sealed plastic Ziploc® bags and then carried out to the laboratory to be analyzed in less than an hour.

After measuring light transmission, the leaf disc was extracted to determine the chlorophyll content according to the standard procedure [5]. Briefly, a 30.5 mm^2^ disc was removed with a paper sheet punch, stripped into smaller pieces with a scalpel and incubated in dimethyl-sulfoxide (Merck 116743) at 65 °C until leaf translucency. In the Chlorophyll-DMSO extract, absorbance readings were taken at 665 and 648 nm with a fiber optic spectrometer FLAME™ (Ocean Optics Inc.). The LCC was calculated according to equation (1) [4].

(1) $$\mathrm{LCC}\left(mg{.cm}^{-2}\right)=7,49A^{664,9}+20,34A^{648,2}$$

**Fig. 4:**
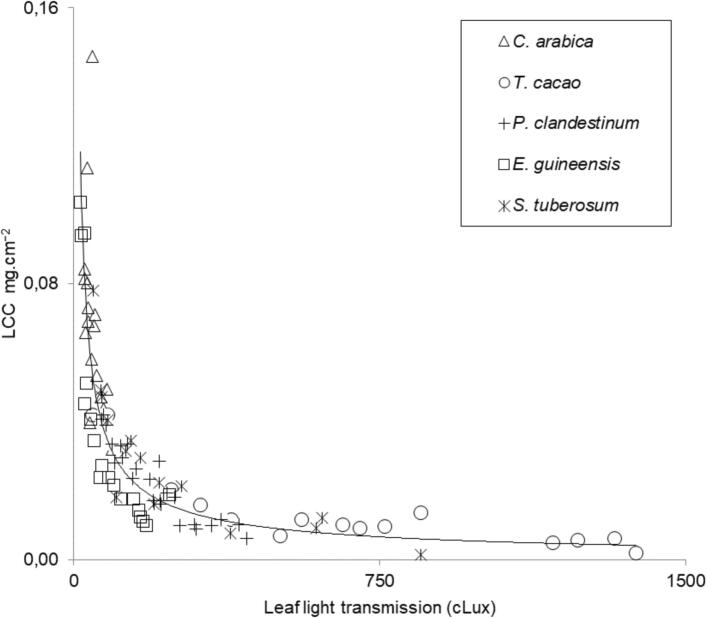
General modelling of leaf chlorophyll estimation from its cLux light transmission values obtained from the smartphone attached Clorofilo accessory. LCC values are laboratory spectrophotometric measurements according to the standard procedure of Hiscox & Israelstam, 1979.

Fig. 4 shows the general association between spectrophotometric chlorophyll content and light transmission readings on leaves. Notable here are both; readings from all species fitting of along a single exponential regression line, as well as the clear difference between subsets of species-specific chlorophyll concentration values. Coffee, palm, cocoa, and potato plants showed highest values, while the lowest values were found in kikuyu grass leaves. A nonlinear regression equation fitted on this data set also showed a high coefficient of determination (R^2^ = 0.829) and a low root mean square error (RMSE = 0.0144). A general estimation of chlorophyll concentration from light transmission values in these plant species would be calculated on equation (2) as follows:

(2) $$\mathrm{LCC}\left(mg{.cm}^{-2}\right)=0,9424\cdot {\mathrm{cLux}}^{-0,748}$$

The results indicate a general strong association between Clorofilo light transmission values and spectrophotometric LCC in these trait dissimilar leaves. These results are comparable to those obtained elsewhere using commercial portable chlorophyll meters like the standard Konica Minolta's SPAD 502™ meter and CCM-200™ from Opti-Sciences [16], [17], [18]. Note that plant group specific chlorophyll content model differences linked to leaf structure were previously presented. By using portable meters Cerovic et al., 2012 found differences between monocots and dicotyledons in their chlorophyll estimation models. This was explained by a larger vascular tissue per unit area for monocots and the largest size of the midrib and cuticle thickness in dicots leaves. Species specific modelling of chlorophyll content from portable meters has been argued by many studies [19], [20]. However, global unified models for multiple species were also proposed [21], [22], [23].

The current global model to estimate chlorophyll content from Clorofilós cLux readings is supported by a coefficient of determination (R^2^ = 0,829) slightly higher than previously presented coefficients from a SPAD-502™ meter study with eight deciduous species, with R^2^ values varying between 0,71–0,72 for linear, quadratic, and exponential regression models [17]. This current model also exceeds the accuracy of overall LCC estimates made in soybean, canola, corn and wheat, in which a highest R^2^ = 0,74 was reached after testing SPAD- 502™, CCM-200™ and Dualex-4™ meters in diverse shape regression models [16].

In conclusion, at least in laboratory settlings this 3D printed device would performs as well as a scientific grade spectrometer to estimate and modelling LCC in these diverse growth form and leaf trait contrasting plant species. Easing the global access to affordable LCC meters would allow a better understanding of general patterning in light transmission by leaves and its association to their most important traits. Although numerous low-cost measurements everywhere are just not possible when more expensive devices are used.

***Comparison of measurements of the Clorofilo accessory to those of a SPAD-502™ meter on coffee* tree leaves**.

On 50 coffee tree leaves, cLux readings from the chlorophyll meter were compared with indices from Konica Minolta's SPAD-502™ meter, which is considered the universal standard for portable chlorophyll meters [6], [21], [22], [24]. The 50 data pairs taken at the same spot on the leaves built a reasonable regression model to match light transmission readings to SPAD index values (R^2^ = 0,92; RMSE = 2,245):

(3) $$\mathrm{cLux}=-34,743ln(SPAD502^{\mathrm{TM}}index)+152,494$$

**Fig. 5:**
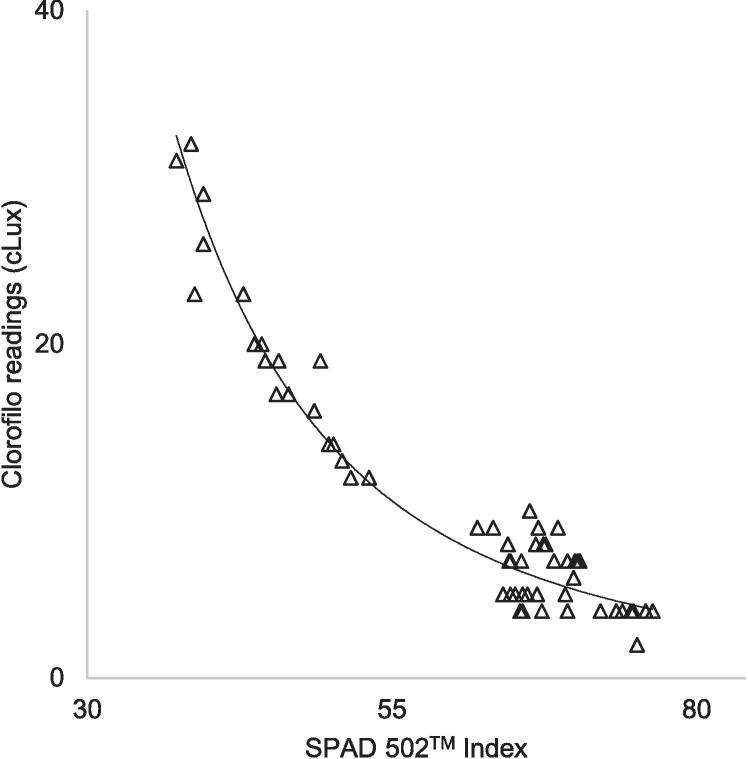
Relationship between SPAD-502™ index meter and cLux values of the Clorofilo accessory.

Fig. 5 shows the scatterplot and logarithmic model line depicted by the regression equation obtained, which suggests a reliable match between both leaf light transmission indexes in the data range studied.

This result shows statistical quality parameters equivalent to those found in studies which compare several commercial meters readings which are supported by similar optical principles such as SPAD 502™, CCM-200™ and atLEAF [19], [23].

The red-light transmission readings of the device presented in this research, like the relative chlorophyll readings from other commercial portable meters such as the SPAD 502™ and the CCM-200™, use mathematical relationships to estimate chlorophyll concentration based on values obtained from traditional analytical methods. A logarithmic model constructed with cLux readings from our chlorophyll meter, compared with SPAD-502™ index measurements in 50 coffee leaves, showed a reliable agreement between both indices. Similar results were found in studies evaluating the performance of commercial meters using similar optical principles, such as the CCM-200™, atLEAF, and SPAD-502™, which is the universal standard device for relative chlorophyll measurements [6, 19 21–24].

### 4.4 Possible further application and limitations

Novel free tools are not without drawbacks. Despite the optical principle of chlorophyll measurement by this accessory is similar to that of other relative chlorophyll measurement technologies, the accuracy of the readings can be affected by device building, environment conditions like lighting and plant properties. In commercial devices like SPAD meters, in natural environments, relative chlorophyll measurements are influenced by physiological phenomena linked to the intracellular distribution of chloroplasts during light exposure [7], [1], such as diastrophe and parastrophe [25]. These phenomena can result in an underestimation of chlorophyll concentration due to the sieve effect or an overestimation of this nutrient due to the detour effect [6].

Also, the mechanical and electrical design is open to changes that will improve this device to exploit the same optical principles to safely measure leaf chlorophyll content as a proxy for plant physiological performance. The actual design needs improvements on its smartphone attachment system to avoid noisy measurements, and its general construction can be enhanced by new materials and simplified assemblage. However, it is considered here that overlooked embedded sensors in smartphones could help make truly deployable tools for open science and sustainable farming, and understanding these sensors need urgent attention.

Some recommendations to improve the device's performance may include the addition of voltage regulators to control the light intensity of the LEDs during prolonged use. This could prevent a decrease in LED power and, consequently, the occurrence of inconsistent measurements. Another alternative could be to correct the cLux readings based on measurements of the LED light intensity without leaf interference before each use. Additionally, it should be noted that the discharge pattern of 3 V lithium batteries from manufacturers typically shows a voltage decay curve with an approximately constant behavior during the first hours of use, which decreases rapidly towards the end of their life, depending on factors such as temperature and discharge current, among others [26] (Seong et al., 2018). Conducting discharge tests of the batteries during prolonged and repetitive measurements can help predict hours of use without measurement variations.

**Fig. 6:**
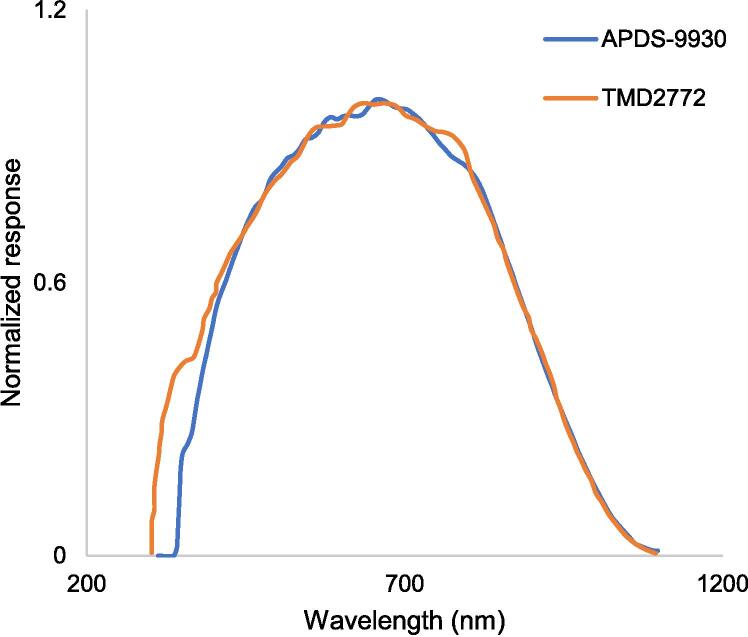
Spectral response of an APDS-9930 sensor embedded on a Sony™ T2 Ultra smartphone, and a TMD2772/TMD2772WA sensor on a Samsung™ Note smartphone, according to the data sheet of the manufacturer (https://datasheetspdf.com/).

Additional experiments were conducted to understand the possibilities and limitations of using outdated or very low-cost smartphones, as these sensors could exhibit differences in response intensity and spectral amplitude. For example, a Sony Xperia T2 Ultra phone includes an APDS-9930 sensor, while a Samsung™ Note phone features a TMD2772/TMD2772WA sensor. For both smartphones the highest spectral response on its sensors (Fig. 6) matches the region with the highest absorption of red light by *a* and *b* chlorophylls [3], [27] and with the peaks of lowest light transmission in the species used in this research (Fig. 8). It is then expected that other types of sensors in smartphones will be useful for chlorophyll determination in plant leaves.

**Fig. 7:**
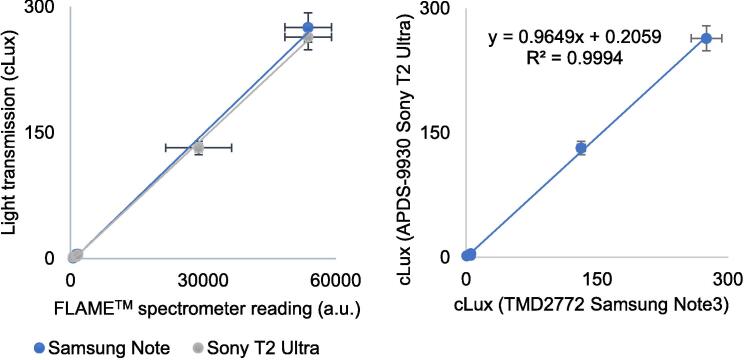
Relative response to 663 nm red light in smartphone and a spectrometer sensors. (a) Relative response in APDS-9930 (Sony™ T2 Ultra) and TMD2772/TMD2772WA (Samsung™ Note 3 phone) sensors as compared to the readings of a FLAME™ spectrometer. (b) Detailed comparative response of the sensors embedded in these dissimilar smartphone brands. Five measurement points were reached by stacking 1 to 5 layers of translucent green cellulose paper. The first tree points in the scatterplot are invisible since their values were below 6 cLux units for the smartphone sensors and 1611 arbitrary units (u.a.) for the FLAME™ spectrometer. (For interpretation of the references to color in this figure legend, the reader is referred to the web version of this article.)

**Fig. 8:**
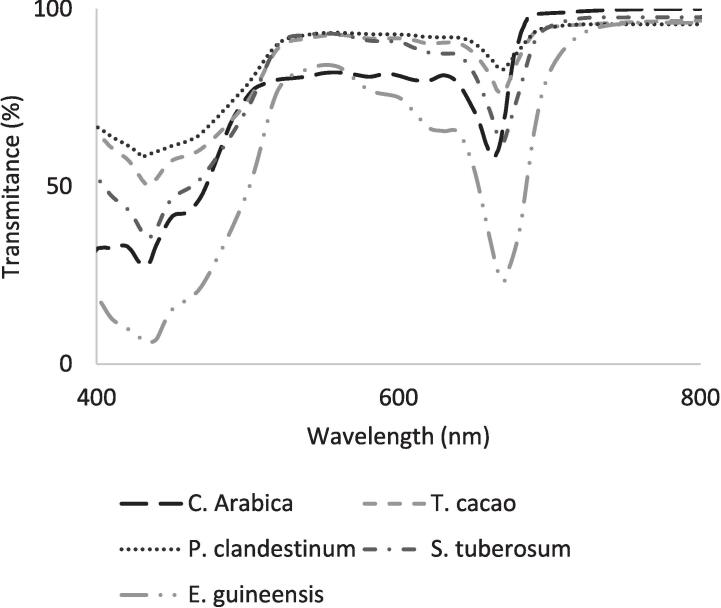
Transmission spectra of chlorophyll extracts in acetone [30] from the leaves of oil palm (Elaeis guineensis; Jacq), potato leaves (Solanum tuberosum; L.), coffee (Coffea arabica L.), cocoa (Theobroma cacao), and kikuyu grass (Pennisetum clandestinum) leaves. .

Source: The authors

An additional experiment tested the specific red-light sensitivity by sensors on outdated smartphones. Readings of a TMD2772/TMD2772WA (Samsung Note™ phone) or APDS-9930 (Sony™ Xperia T2 Ultra phone) were compared with a FLAME™ (Ocean Insight) spectrometer measurements in a test which measured the intensity transmission of red-light when passing through 1 to 5 successive layers of translucent green cellulose paper (cellophane). It was used here as a proxy for green interference red filtering, which partially restricts the passage of the red LED light used by Clorofilo. Translucent cellulose paper (cellophane) acts as an optical filter to analyze the influence of specific bands of light on the development of plants and on applications with light transmission [28], [29]. Here it is a uniform material which regulates red light band intensity transmission to quantitatively relate sensors sensitivity, without the hassles of natural variations in plant leaves [22].

Fig. 7 shows that the response of these sensors linearly relates to the response of the FLAME™ spectrometer (Ocean Insight, FL, USA) readings, even though their dynamic range is quite different. The maximum 300 lx light intensity values on these smartphones are very small compared to the 0 to 53,736 light intensity range of the FLAME™ spectrometer. It would explain the higher resolution of the spectrometer's measurements and their ability to exhibit small differences in light intensity and its associated properties.

This test shows differences in the dynamic range of different smartphones sensors, and suggests the need to standardize measurements on a common scale if different smartphones are used. However, the linear response between these different sensors suggests that multiple but standardized chlorophyll measurements in leaves of plants affected by sanitary or nutritional conditions must be similar, regardless of the phone or device used.

Even though those differences among smartphone manufacturers sensors ALS sensors, these results may go beyond the local exploration of chlorophyll accumulation by leaves of some tropical crops, and suggest further possibilities for the wide use of sensors in smartphones by scientists and farmers. A speculation just to explore: due to the ubiquitous presence of GPS sensors and internet connection in smartphones today, the simultaneous calibrated accessorizing of ALS and GPS sensors may lead to develop smartphone powered precision agriculture tools, such as on field georeferenced mapping of plant nutritional and health status. It is possible since there was a strong correlation of Clorofilo readings to SPAD 502™ index. These meters are also widely tested to measure leaf chlorophyll and plant nitrogen content. A logical consequence of adapting and implementing this type of tools is that it would be possible for the user to discover useful geographical patterns of plant status variations in agricultural fields in case these exist.

### 4.5 Ethics statements

The authors declare that they have no known competing financial interests or personal relationships that could have appeared to influence the work reported in this paper.

### CRediT authorship contribution statement

**Karen Ospino-Villalba:** Writing – original draft, Validation, Investigation, Funding acquisition, Formal analysis. **Daniel Gaviria:** Resources, Methodology, Conceptualization. **Daniel Pineda:** Resources, Methodology, Conceptualization. **Juan Pérez:** Supervision, Writing – review & editing, Conceptualization, Methodology, Formal analysis.

## Declaration of competing interest

The authors declare that they have no known competing financial interests or personal relationships that could have appeared to influence the work reported in this paper.
